# Potential for quantifying general environmental resilience of dairy cattle in sub-Saharan Africa using deviations in milk yield

**DOI:** 10.3389/fgene.2023.1208158

**Published:** 2023-12-15

**Authors:** Richard D. Oloo, Raphael Mrode, Jörn Bennewitz, Chinyere C. Ekine-Dzivenu, Julie M. K. Ojango, Gebregziabher Gebreyohanes, Okeyo A. Mwai, Mizeck G. G. Chagunda

**Affiliations:** ^1^ Animal Breeding and Husbandry in the Tropics and Subtropics, University of Hohenheim, Stuttgart, Germany; ^2^ Livestock Genetics, International Livestock Research Institute, Nairobi, Kenya; ^3^ Animal and Veterinary Science, Scotland Rural College, Edinburgh, United Kingdom; ^4^ Animal Genetics and Breeding, University of Hohenheim, Stuttgart, Germany

**Keywords:** resilience indicators, robustness, cow, milk production, log-transformed variance, African tropics

## Abstract

**Introduction:** Genetic improvement of general resilience of dairy cattle is deemed as a part of the solution to low dairy productivity and poor cattle adaptability in sub-Saharan Africa (SSA). While indicators of general resilience have been proposed and evaluated in other regions, their applicability in SSA remains unexplored. This study sought to test the viability of utilizing log-transformed variance (LnVar), autocorrelation (r_auto_), and skewness (Skew) of deviations in milk yield as indicators of general resilience of dairy cows performing in the tropical environment of Kenya.

**Methods:** Test-day milk yield records of 2,670 first-parity cows performing in three distinct agroecological zones of Kenya were used. To predict expected milk yield, quantile regression was used to model lactation curve for each cow. Subsequently, resilience indicators were defined based on actual and standardized deviations of observed milk yield from the expected milk yield. The genetic parameters of these indicators were estimated, and their associations with longevity and average test-day milk yield were examined.

**Results:** All indicators were heritable except skewness of actual and standardized deviation. The log-transformed variance of actual (LnVar1) and standardized (LnVar2) deviations had the highest heritabilities of 0.19 ± 0.04 and 0.17 ± 0.04, respectively. Auto-correlation of actual (r_auto_1) and standardized (r_auto_2) deviations had heritabilities of 0.05 ± 0.03 and 0.07 ± 0.03, respectively. Weak to moderate genetic correlations were observed among resilience indicators. Both r_auto_ and Skew indicators had negligible genetic correlations with both longevity and average test-day milk yield. LnVar1 and LnVar2 were genetically associated with better longevity (rg = −0.47 ± 0.26 and −0.49 ± 0.26, respectively). Whereas LnVar1 suggested that resilient animals produce lower average test-day milk yield, LnVar2 revealed a genetic association between resilience and higher average test-day milk yield.

**Discussion:** Log transformed variance of deviations in milk yield holds a significant potential as a robust resilience indicator for dairy animals performing in SSA. Moreover, standardized as opposed to actual deviations should be employed in defining resilience indicators because the resultant indicator does not inaccurately infer that low-producing animals are inherently resilient. This study offers an opportunity for enhancing the productivity of dairy cattle performing in SSA through selective breeding for resilience to environmental stressors.

## 1 Introduction

General environmental resilience (simply put as general resilience) of an animal is its capacity to be either minimally affected by an environmental disturbance or rapidly return to the behavioral, physiological, cognitive, health, affective, and production states pertained before exposure to a disturbance (Colditz and Brad, 2016). Resilience is different from resistance, which is the ability of the host animal to exert control over the disturbance (Bishop, 2012). Whereas resistance is disturbance-specific, general resilience combines all forms of disturbances the animal is exposed to (Colditz and Brad, 2016; Friggens et al., 2017; Berghof et al., 2019b). The idea of resilience compares the differences in the measurement of phenotypes among individuals after exposure to environmental challenges (Rutter, 2012) and it arises because of better adaptability or lower sensitivity to a challenging state of affairs. The performance of a more resilient animal need not be the same as without a disturbance, but rather, the negative shift in its performance should be relatively low compared to a less resilient individual performing in the same conditions. Resilience is closely related to but different from robustness, the ability of an animal to express its production potential in a wide range of environmental conditions without compromising its reproduction, health, and wellbeing (Knap, 2005; Colditz and Brad, 2016). The disturbances associated with general resilience are normally short-term, situation-specific, episodic, sporadic, and non-permanent attributes of the environment that affect only a few individuals within the environment. The perturbations related to robustness are by and large long-term and persistent or cyclical characteristics of the environment that affect the entire population (Strandberg, 2009; Colditz and Brad, 2016).

Dairy production in sub-Saharan Africa (SSA) is not sufficient to cater for the regional dairy needs and as a result, pressure for increased milk production is still building. However, with climate change and its negative impacts on the environment, it is necessary to shift the focus of dairy production from simply increasing production to prioritizing efficiency and sustainability of milk production (Hernández-Castellano et al., 2019; Oloo et al., 2022b). Sustainable dairy production practices that guarantee food security for the growing population, while mitigating the adverse effects of climate change, need to be adopted. The dairy production environment of SSA is confronted with numerous environmental perturbations that negatively affect the performance of dairy animals (Thornton et al., 2009; Nardone et al., 2010; Hernández-Castellano et al., 2019). Most of these disturbances are naturally occurring and cannot be averted through husbandry interventions. Therefore, it is vital to breed for resilience to improve the genetic potential of animals to weather environmental stressors and maintain their optimal production levels.

In breeding for resilience to environmental disturbances, appropriate ways of quantifying resilience need to be defined. However, because functional traits related to resilience are difficult to measure, quantifying resilience has been a challenge, especially in SSA (Oloo et al., 2023). Empirical indicators of resilience based on deviations from the expected performance have been defined by Berghof et al. (2019b) based on proposals made by Scheffer et al., 2015, Scheffer et al. 2018, Scheffer et al. 2009) on the quantification of resilience. These indicators include variance of deviations, autocorrelation of deviations, and skewness of deviations. They use longitudinal performance data of the animals to capture general resilience. Resilient animals are expected to have fewer and smaller deviations since they are less influenced by the disturbances than non-resilient animals. Under the assumption that the environmental disturbance reduces the phenotype value as in the case of milk yield, resilient genotypes have a low variance of deviations, low autocorrelation of deviations, and higher skewness of deviations. The opposite is the case for non-resilient animals (Scheffer et al., 2018; Berghof et al., 2019b). These indicators display genetic variation and are genetically favorably correlated with health and fitness traits (Berghof et al., 2019a; Poppe et al., 2020; Poppe et al., 2021a). Another potential indicator of resilience is the variance of lactation milk yield, where low variance indicates better resilience. It is moderately heritable and is genetically associated with better longevity, better udder health, and reduced ketosis (Elgersma et al., 2018; Poppe et al., 2020).

The potential of using these indicators to quantify and measure resilience remains untested in SSA. Applying conclusions and recommendations made on livestock resilience from temperate countries in tropical SSA is not very feasible for several reasons. The majority of dairy cattle in SSA are crossbreeds of different proportions of zebu and taurine breeds (Ojango et al., 2017; Oloo et al., 2022a; Habimana et al., 2023), which are quite different from the cattle bred in developed countries in terms of body conformation and size, performance, and feed requirement. In addition, the environmental stressors affecting dairy cattle and the level of animal husbandry and herd management practices in the SSA are disparate from those in the temperate world (Oloo et al., 2023). Besides, precision livestock farming technologies used in developed countries are yet to be witnessed in most farms in SSA, hence performance data kept on animals are either unavailable or scanty (Mrode et al., 2020). Therefore, the degree of resilience of dairy cattle in SSA should be quantified based on the cattle genotypes reared in SSA, available data, and the set of disturbances to which they are exposed.

Previous studies have used actual deviations to derive resilience indicators (Berghof et al., 2019a; Poppe et al., 2020; Poppe et al. 2021a; Poppe et al. 2021b). However, this would only apply if the animals were of the same genotype with similar production potential and under similar management conditions. Otherwise, it would tend to categorize low-producing animals as being more resilient. For instance, a deviation of 3 kg from an expected value of 5 kg is equivalent to 60% while the same deviation from an expectation of 20 kg is equivalent to only 15%. The use of actual deviations would conclude that these animals have the same deviation and hence, are similarly affected by disturbances which is not true. Therefore, it would be interesting to use both actual and standardized deviations to derive resilience indicators and compare their genetic parameters. The objectives of this study thus were: i) to investigate genetic parameters of resilience indicators derived from both actual and standardized deviations using test-day milk records of dairy cattle of different breeds performing in three different agro-ecological zones of Kenya; ii) to investigate the relationship between different resilience indicators and longevity and average test-day milk yield.

## 2 Materials and methods

Data used in this study are from dairy cows in three different herds, situated in different agroecological zones of Kenya. Two of the herds were performing in the agroecological zone IV (semi-arid) but in regions of the country where different agricultural practices were predominant. The agricultural practices adopted in the region were thus used to classify the farms as semi-arid arable (SAA) and semi-arid-pasture based (SAP). The third farm was in agroecological zone V (semi-humid (SH)).

### 2.1 Climatic conditions of the agroecological zones

The SAA herd receives annual average rainfall ranging between 800 and 950 mm. It has a bimodal rainfall pattern, with the first rainy season occurring from March to April and the second season from November to December. The rainfall is sufficient for regeneration of annual/perennial pasture and browse material to support livestock production. The mean daily temperature ranges from 20.2°C to 24.6°C (Jaetzold et al., 2006; Makueni County, 2013). The SAP zone has an annual mean temperature ranging between 18.3°C and 19.6°C and an annual average rainfall of 650–750 mm. It receives bimodal rainfall, with a main rainy season occurring from March to June and a short rainy season from October to December. Droughts are sometimes experienced in this agroecological zone, of which the frequency and severity have been on the rise in recent times due to climate change (Jaetzold et al., 2010b). Water in this zone is not sufficient to support sustainable crop production, thus, farmers mainly practice livestock rearing. The mean daily temperature of the SH zone ranges from 25.2°C to 27.0°C and the average rainfall from 850 to 1100 mm. It has two rainy seasons with long rain occurring between April to June and short rains between October to December. The region depends mostly on the long rains to support animal production in ranches and some crop production during the short rainy season. Rainfall received is adequate for regeneration of enough annual/perennial pasture and browse material to support livestock until the following rain season (Malindi District, 2008; Jaetzold et al., 2010a).

### 2.2 Herd management practices

In the SAA herd, calves are separated from their dams at 5 days of age and tube-fed on fresh milk twice a day until they are 2 months old. They are then weaned from dairy meal to hay, concentrates (wheat bran and maize germ), and mineral salt licks that are provided *ad libitum*. At the age of 9 months, they are moved into the grazing herd, where they feed on natural pastures supplemented with mineral licks. Bulls and heifers are fed on natural pastures with mineral lick given as supplements. The lactating herd is grazed on natural pastures except during milking time when they are provided with a mixture of seed cake, wheat bran, and maize germ. Milking is done by hand twice a day. During the dry season, when there is little standing hay and no preserved hay, the animals are fed on silage made from natural grass as supplements. Water is availed to all animal groups *ad libitum*. The animals are drenched regularly until they are 9 months old after which it is stopped, and the animals are then treated only when there is an infection. Ectoparasites are controlled in all animal groups by dipping on a weekly basis. Vaccination is done routinely against Lumpy Skin Disease (LSD), foot-and-mouth disease (FMD), Rift Valley Fever (RVF), Brucellosis, Anthrax, Black-quarter, and Bovine Viral Diarrhea (BVD).

In the SAP farm, calves are separated from their dams within 12 h after birth, and thereafter bucket-fed on colostrum for the first 4 days of life and on fresh milk for about 9 weeks. They are gradually weaned on concentrates and hay starting from 5 weeks of age and are fully weaned from whole milk when they are 9 weeks of age. This is followed by rearing on natural pastures except under drought conditions when they receive lucerne hay as supplements. Bulls and heifers are migrated to separate farms where they are grazed on natural pastures with no supplementary feeding for their remaining rearing period. They, however, have access to a balanced mineral salt lick. Lactating cows are grazed on the best pastures using a planned rotational grazing system, with the cows rarely staying longer than a week in one paddock. The cows are milked twice daily using machines in a modern milking parlor. Water is made available to all animal groups *ad libitum*. All animals are treated for endoparasites and dipped at least once weekly to control ectoparasites. Routine vaccination is done against FMD, rinderpest, Black-quarter, Anthrax, RVF, and brucellosis.

In the SH herd, calves are removed from their dams within 1 hour after birth and bucket-fed twice a day with colostrum for the first 3 days and milk up to 4 months of age. Additionally, they receive calf early weaner pellets and total mixed ration (TMR) silage *ad libitum* as a dairy meal replacement. The pellets and TMR are gradually replaced with sifted chicken litter/maize bran mixture and normal silage (made from standing hay or maize stover), respectively. Heifers are fully zero-grazed until 15 months when they join the adult herd. The heifers (15 months of age and older) are grazed on natural pasture and paddocked together as a single herd. Lactating cows are zero-grazed and additionally provided with TMR *ad libitum*. Milking cows are hand-milked twice or three times a day depending on the milk market situation. Water is availed to all the animals *ad libitum*. Ticks and flies are controlled by pour-on 1–2 weeks apart depending on the tick and biting fly challenge. No deworming is done for any age group. Vaccination against FMD, LSD, RVF, Brucellosis, Rabies, Anthrax, Black-quarter, and BVD is done routinely.

### 2.3 Data

Data of first parity dairy cows born between 1980 and 2019 were assessed for quality and used in this study. Only cows with age at first calving between 21 and 60 months were used. The lower limit was based on the possibility of including abortions that occurred in late pregnancy. The upper limit took care of the likelihood of a subsequent calving event being misclassified as the first calving due to unrecorded first calving. Milk yield values below 0.5 and above 45 kg were excluded following recommendations by Mrode et al. (2021), and all milk yield records were used up to 400 days after calving. The standard limit of 305 days in milk does not reflect the real production patterns of the population studied, because, in the study areas, farmers tend to milk their cows longer, upon consecutive failure of cows to conceive in time due to malnutrition-triggered infertility or poor estrus detection or both (Mrode et al., 2021). Preliminary analyses showed that there were cows with lactation lengths that were above 400 days, signifying a possibility of unrecorded calving events in between, thus erroneously placing new lactation records in the previous ones. To have enough records for modeling lactation curves, each cow was required to have 10 or more test-day milk records. The clean data was first used to model lactation curves and later define resilience indicators.

### 2.4 Fitting lactation curves

Lactation curve for each cow based on test-day milk yield was fitted to predict the expected milk yield of a cow on each day in the absence of disturbances. The deviations in milk production from such a curve were used to indicate the level of resilience. A fourth-order polynomial quantile regression model defined below was used to compute the expected milk yield for each cow.

$${yield}_{t}=\beta_{0}+\beta_{1}t+\beta_{2}t^{2}+\beta_{3}t^{3}+\beta_{4}t^{4}+\varepsilon t$$

where ${yield}_{t}$ is the observed milk yield on t^th^ days in milk (DIM *t*) and *ε* is the error term.

**FIGURE 1:**
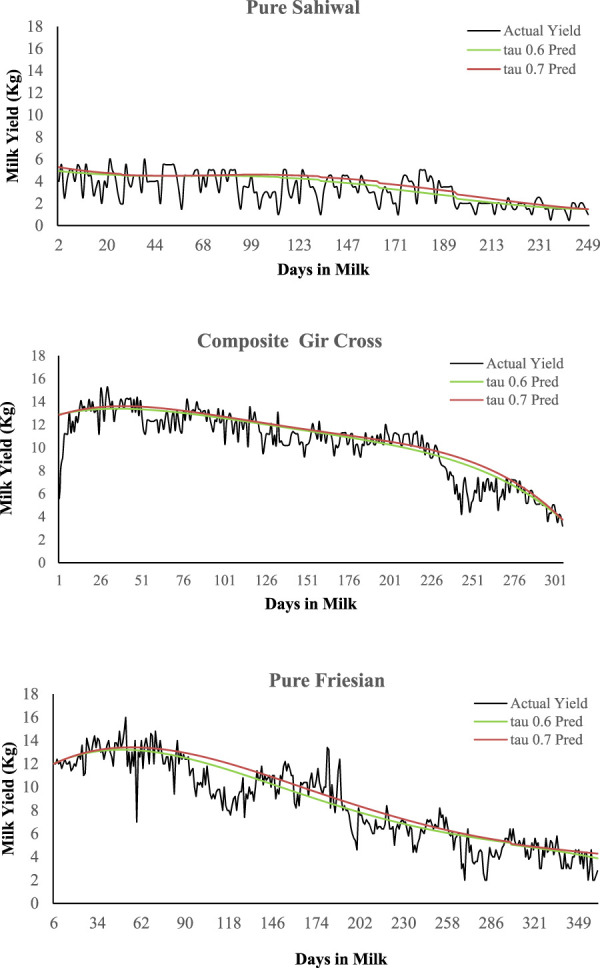
Examples of actual and modeled lactation curves of animals of different breed groups. Lactation curves were modeled using quantile regression method. Two quantiles, 0.6 (tau 0.6 Pred) and 0.7 (tau 0.7 Pred) were tested before settling on quantile 0.7 upon visual inspection.

0.6^th^ and 0.7^th^ quantiles were first tested (Figure 1) before settling on 0.7^th^ quantile upon visual inspection of the modeled curves of random animals vis-à-vis their actual production trend.

### 2.5 Defining resilience indicators

The observed milk production and expected milk yield from the lactation curves were used to calculate actual and standardized deviation of *j*th animal on *i*th test-day as shown below:

$${Actual\;Deviation}_{ij}={\mathrm{Observed}\;Milk\;Yield}_{ij}-\;{Expected\;Milk\;Yield}_{ij}$$

$${Standardized\;Deviation}_{ij}=\frac{{\mathrm{Observed}\;Milk\;Yield}_{ij}-\;{Expected\;Milk\;Yield}_{ij}}{{Expected\;Milk\;Yield}_{ij}}$$

Both actual and standardized deviations were used to calculate three resilience indicators: variance (LnVar), lag-1 autocorrelation (r_auto_), and skewness (Skew) of deviations. Log-transformation of variance of deviation was necessary to make the trait assume a normal distribution. The indicators derived from actual deviations were termed LnVar1, r_auto_1, and Skew1 while those derived from standardized deviations were denoted LnVar2, r_auto_2, and Skew2.

The LnVar of the *t* ^he^ individual was calculated as:

$$\ln\left({variance}_{j}\right)=\ln\left(\frac{\sum_{i=1}^{n_{j}}{\left(x_{ij}-{\bar{x}}_{j}\right)}^{2}}{n_{j}-1}\right)$$

where *x*_*ij*_ is deviation *i* of the j^th^ individual, $\bar{x}$ _j_ is the mean of deviations of the *j* ^*th*^ individual, and *n*_*j*_ is the number of deviation observations of the *j* ^*th*^ individual.

The r_auto_ of deviations of the *j* ^th^ individual was calculated as:

$${autocorrelation}_{j}=\frac{\sum_{i=1}^{n_{j}-1}\left(x_{ij}-{\bar{x}}_{j}\right)\left(x_{\left(i+1\right)j}-{\bar{x}}_{j}\right)}{\sum_{i=1}^{n_{j}}{\left(x_{ij}-{\bar{x}}_{j}\right)}^{2}}$$

where *n*_*j*_ is the number of pairs of subsequent deviation observations of the *j* ^*th*^ individual, *x*_*ij*_ is deviation *i* of the j^th^ individual, $\bar{x}$ _j_ is the mean of deviations of the *j* ^*th*^ individual, and *x*_*(i+1)j*_ is the subsequent deviation of deviation *i* of the j^th^ individual.

The skew of deviations of the *j* ^th^ individual was calculated as:

$${skew}_{j}=\frac{n_{j}}{\left(n_{j}-1\right)\left(n_{j}-2\right)}\sum_{i=1}^{n_{j}}{\left(\frac{x_{ij}-{\bar{x}}_{j}}{\sqrt{s_{j}^{2}}}\right)}^{3}$$

where $n_{j}$ is the number of deviation observations of the *j* ^*th*^ individual, $x_{ij}$ is deviation *i* of the *j* ^*th*^ individual, ${\bar{x}}_{j}$ is the mean of deviations of the *j* ^*th*^ individual, and $s_{j}^{2}$ is the variance of deviations of the *j* ^*th*^ individual.

### 2.6 Data editing

**FIGURE 2:**
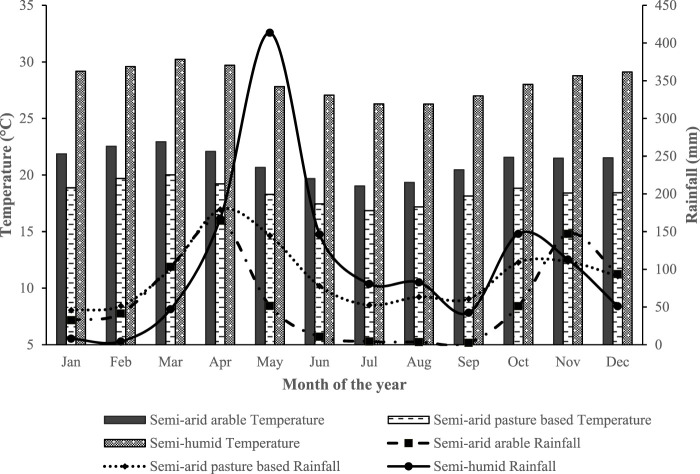
Average monthly rainfall and temperature of the agroecological zones (based on global climate data from January 2007 to December 2019 extracted from aWhere online climate website) from where the herds studied came.

Measurement of resilience indicators of individual cows that deviated more than 4 standard deviations (SD) from the mean was set to missing. Using climatic data extracted from aWhere (www.awhere.com) and the available recent classification of climatic seasons in the literature (Jaetzold et al., 2006; Malindi District, 2008; Jaetzold et al., 2010b; Makueni County, 2013), four seasons were defined for each zone based on the rainfall pattern. The long rain and short rain periods were considered green seasons 1 and 2, respectively. The dry period before the long rain was considered as dry season 1 and that before the short rains as dry season 2. The seasons of each agroecological zone with rainfall and temperature patterns are presented in Table 1; Figure 2. To correct for season and year of calving, a contemporary grouping of year-season (YS) was done with 40 possible years of calving (1982–2021) and 4 possible seasons. YS groups with less than 5 lactations were excluded from the analysis. After editing the data, 2,670 resilience data for 2,670 cows were used for the analysis. These animals were grouped into three breed groups based on the proportion of taurine genetics in their breed composition as provided by the farmer: breed group 1 (BG1) (≤50% *Bos taurus*, n = 928), BG2 (>50–87.5% *Bos taurus*, n = 598 and BG3, >87.5–100% *Bos taurus*, n = 1,144). The dairy breeds included Holstein Friesian, Jersey, Guernsey, Ayrshire, Brown Swiss, Fleckvieh, Milking Shorthorn, Meuse Rhine Issel, and Montbéliarde. Zebu breeds in this population included Sahiwal, Boran, and Gir.

**TABLE 1:** Classification of climatic seasons of the three agroecological zones where the animals under study came from.

Agroecological zone	Seasons as defined by months of the year			
	Dry season 1	Green season 1	Dry season 2	Green season 2
Semi-arid arable (SAA)	January to February	March to April	May to October	November to December
Semi-arid pasture (SAP)	January to February	March to June	July to September	October to December
Semi-humid (SH)	December to March	April to June	July to September	October to November

### 2.7 Statistical analysis

#### 2.7.1 Fixed effect factors of variation

A fixed effect linear model shown below was first fitted to determine non-genetic factors that significantly affect individual measurements of the resilience phenotype.

$$y_{ijklmno}=U+{\mathrm{breed}}_{i}+{\mathrm{env}}_{j}+{\left(\mathrm{breed}*\;\mathrm{env}\right)}_{ij}+{\mathrm{ysc}}_{k}+{\mathrm{age}}_{l}+{\mathrm{Obs}}_{m}+{\dim1}_{n}+{\dim2}_{n}+e_{ijklmno}$$

where $y_{ijklmno}$ is the vector for individual resilience indicator measurement for *o* ^*th*^ animal, *U* corresponds to the population mean, ${breed}_{i}$ is the *i* ^*th*^ breed group (i = 1–3), ${env}_{j}$ is the environment which combines climatic conditions and herd management (j = 1–3), ( $\text{breed}*\;{\left.env\right)}_{ij}$ is the interaction term between *i* ^th^ genotypic class and *j* ^th^ environment, ${ysc}_{k}$ is the *k* ^*th*^ year-season of calving (k = 1–137), ${age}_{l}$ represent *l* ^*th*^ age at first calving in months (l = 21–60), ${Obs}_{m}$ is the number of milk records used to calculate the resilient indicator (m = 10–400) $,{\;\dim1}_{n}$ and ${\dim2}_{n}$ are the first and the last DIM classes of the DIM of *o*_*th*_ cow (n = 1–10), respectively and $e_{ijklmno}$ is the residual error. Least-square means (LSM) of different breed groups and environments were calculated and contrasted.

#### 2.7.2 Genetic parameters of resilience indicators

A univariate animal model shown below was used to estimate (co) variance of all the resilience indicators and average test-day milk yield using ASReml-R 4.1 (Gilmour et al., 2015):

y=Xβ+Za+e

where ***y*** is a measurement of individual phenotype for the resilience trait, ***β*** is the solutions of the fixed effects in the model which included genotypic class, the environment, (which accounted for the confounded effects such as herd management practices), year-season of calving, calving age in months first and last class of days in milk and total number of milk observations used to derive resilience indicator. ***a*** is the solutions of random cow additive genetic effects and ***e*** is the vector of random residual effects. The vectors of random animal effects ***a*** and residual effects ***e*** were assumed to follow normal distributions with ***a*** ∼ *N (0;* ${A\sigma }_{a}^{2}$ *)* and ***e*** ∼ *N (0;* ${I\sigma }_{e}^{2}$ *)*, where ***A*** corresponds to the numerator relationship matrix, ***I*** correspond to the identity matrix, $\sigma_{a}^{2}$ is the additive genetic variance, and $\sigma_{e}^{2}$ is the residual variance. ***X*** is the incidence matrix relating observations to fixed effects; ***Z*** is the incidence matrix relating records to random animal effects. The pedigree used to construct the numerator relationship matrix consisted of 4,933 individuals.

Heritabilities were calculated as $h^{2}=\frac{\sigma_{a}^{2}}{\sigma_{p}^{2}}$ where $\sigma_{a}^{2}$ and $\sigma_{p}^{2}$ are additive and phenotypic variance, respectively and $\sigma_{p}^{2}$ is the sum of additive ( $\sigma_{a}^{2}$ ) and residual ( $\sigma_{e}^{2}$ ) variances. The likelihood ratio test was used to test whether the estimated heritabilities were significantly different from zero under the assumption that the likelihood ratio follows a $\chi^{2}$ distribution. The tested model was compared against a model without animal effects. The likelihood ratio test was -2ln (∧(y)) with $\wedge \left(y\right)=\frac{\max\;\left[L_{0}\left|y\right.\right]}{\max\;\left[L_{1}\left|y\right.\right]}$ where $L_{0}$ is the likelihood under the null hypothesis with animal effects excluded, $L_{1}$ is the likelihood under the alternative hypothesis with animal effects included in the tested model and y is the given dataset.

The genetic coefficient of variation (GCV) was calculated as $GCV=\frac{\sqrt{\sigma_{a}^{2}}}{µ}$ , where $\sigma_{a}^{2}$ is the additive genetic variance of the resilience indicator and µ is the overall mean of that resilience indicator. However, the GCV of indicators based on variance was calculated as $\sqrt{\sigma_{a}^{2}}$ because these indicators had already been log-transformed. Log transformation assumes an exponential model thus $\sqrt{\sigma_{a}^{2}}$ does not have units and division by overall mean brings about redundancy.

Phenotypic and genetic correlations between the different resilience indicators, and between the resilience indicators and average test-day milk yield, were estimated using variances and covariances estimated from the following bivariate mixed animal model:

$$\left[\begin{array}{c} y_{1}\\ y_{2} \end{array}\right]=\left[\begin{array}{cc} X_{1} & 0\\ 0 & X_{2} \end{array}\right]\left[\begin{array}{c} b_{1}\\ b_{2} \end{array}\right]+\left[\begin{array}{cc} Z_{1} & 0\\ 0 & Z_{2} \end{array}\right]\left[\begin{array}{c} a_{1}\\ a_{2} \end{array}\right]+\left[\begin{array}{c} e_{1}\\ e_{2} \end{array}\right]$$

where $y_{i}$ is a vector with observations on trait *i*; $b_{i}$ is a vector with the fixed effects for trait *i*, which were the same as in the univariate analysis; $a_{i}$ is a vector with the additive genetic effects for trait *i*; and $e_{i}$ is a vector with the residuals for trait *i*; $X_{i}$ and $Z_{i}$ are incidence matrices linking the records in $y_{i}$ to the fixed effects and additive genetic effects, respectively. The additive genetic effects for all traits were assumed to follow a normal distribution with a mean of 0, a genetic variance of $\sigma_{a_{i}}^{2}$ for trait *i*, and a genetic covariance of $\sigma_{a_{1}a_{2}}$ : $\left[\begin{array}{c} a_{1}\\ a_{2} \end{array}\right]$ ∼ *N* $\left[\left(\begin{array}{c} 0\\ 0 \end{array}\right),A⊗\left[\begin{array}{cc} \sigma_{a_{1}}^{2} & \sigma_{a_{1}a_{2}}\\ \sigma_{a_{1}a_{2}} & \sigma_{a_{2}}^{2} \end{array}\right]\right]$ . The residuals were assumed to be normally distributed with a mean of 0, a residual variance of $\sigma_{e_{i}}^{2}$ for trait *i*, and a residual covariance between $\sigma_{e_{1}e_{2}}$ **:** $\left[\begin{array}{c} e_{1}\\ e_{2} \end{array}\right]$ ∼ ***N*** $\left[\left(\begin{array}{c} 0\\ 0 \end{array}\right),I⊗\left[\begin{array}{cc} \sigma_{e_{1}}^{2} & \sigma_{e_{1}e_{2}}\\ \sigma_{e_{1}e_{2}} & \sigma_{e_{2}}^{2} \end{array}\right]\right]$ .

Likelihood ratio test was used to determine whether genetic correlations among resilience indicators were significantly different from zero, by comparing the modeled equation to a bivariate model with additive genetic covariance fixed at zero.

#### 2.7.3 Resilience indicators and longevity traits

To examine the relationship between each resilience indicator and longevity, phenotypic and genetic correlations between them were estimated. Two functional longevity traits, namely, productive lifespan and herd life, were used as measures of longevity. Productive life span was defined as a difference in days between the first calving date and date of exit and herd life as the age of an animal in days before it exited the herd (Ghaderi-Zefrehei et al., 2017). The exit reason was restricted to only death from a disease or disposal for slaughter. A total of 1,129 exit records were used for longevity analyses. Bivariate analyses were used for this purpose using the linear animal models for resilience indicators and the following linear models for productive life span and herd life:

$${\mathrm{pls}}_{abcdefghj}=U+{\mathrm{breed}}_{a}+{\mathrm{env}}_{b}+{\mathrm{ysb}}_{c}+{\mathrm{lactations}}_{d}+{\mathrm{exitcode}}_{e}+{\mathrm{yse}}_{f}+{\mathrm{afc}}_{g}+{\mathrm{AMY}}_{h}+a_{j}+e_{abcdefghj}$$

$${\mathrm{hl}}_{abcdefgj}=U+{\mathrm{AMY}+\mathrm{breed}}_{a}+{\mathrm{env}}_{b}+{\mathrm{ysb}}_{c}+{\mathrm{lactations}}_{d}+{\mathrm{exitcode}}_{e}+{\mathrm{yse}}_{f}+{\mathrm{AMY}}_{h}+a_{j}+e_{abcdefhj}$$

where ${\mathrm{pls}}_{abcdefgj}$ and ${\mathrm{hl}}_{abcdefj}$ are the productive lifespan and herd life of animal j, respectively, ${breed}_{a}$ is the fixed effect of the breed group of the animal (a = 1–3), ${env}_{b}$ is the fixed effect of the environment (agroecological zone) where the animal was reared (b = 1–3), ${ysb}_{c}$ is the fixed effect of the year season of birth (c = 1–98), ${lactations}_{d}$ is the fixed effect of the total number of calving before exit (d = 1–14), ${exitcode}_{e}$ is the fixed effect of the exit reason for the animal, (e = 1–2) and ${yse}_{f}$ is the fixed effect of the year season of exit (f = 1–83), ${afc}_{g}$ is the fixed effect of the age at first calving in months (g = 21–60), ${\text{AMY}}_{h}$ is the average test-day milk yield, and $a_{j}$ is the random additive genetic effect of the j^th^ animal assumed to be ∼ *N (0;* ${A\sigma }_{a}^{2}$ *)*, and $e_{abcdefghj}$ and $e_{abcdefgj}$ are the residual terms of productive lifespan and herd life, respectively assumed to be ∼*N (0;* ${I\sigma }_{e}^{2}$ *)*. Assumed (co)variance structures of the random terms of the model were ${A\sigma }_{a}^{2}$ and ${I\sigma }_{e}^{2}$ where ***A*** corresponds to the numerator relationship matrix, ***I*** corresponds to the identity matrix, $\sigma_{a}^{2}$ is the additive genetic variance, and $\sigma_{e}^{2}$ is the residual variance. The pedigree used to construct the numerator relationship matrix consisted of 4,506 individuals. The significance of genetic correlations was tested as described in Section 2.7.2 above. Fisher’s r-to-z-transformation was used to test whether phenotypic correlations were significantly different from zero using the following test statistic:

$$z=0.5\;\ln\frac{\left(1+r\right)}{\left(1-r\right)}$$

where r is the estimated phenotypic correlation, and z follows a normal distribution with standard deviation 1/√(n-3), where n is the sample size.

## 3 Results

### 3.1 Descriptive statistics of the resilience indicators

Table 2 presents summary statistics of the resilience indicators and average milk yield. The total number of animals used to analyze each indicator differed due to differences in the number of outliers excluded from the analysis. The animals varied greatly in the quantification of their resilience indicators, as signified by a high coefficient of variations (CV). Skew2, Skew1, and LnVar1 had the widest range of values, whereas LnVar2 and average test-day milk yield (AMY) were the least distributed. It is worth noting that the means of most indicators were close to zero because of the presence of both negative and positive values in their distribution and this might have inflated their CV. r_auto_1 and r_auto_2 were almost identical and had a similar distribution.

**TABLE 2:** Descriptive statistics of resilience indicators based on daily milk yield (LnVar: variance of deviations, RawVar: variance of raw milk yield, Skew: skewness of deviations, r_auto_: lag-1 auto-correlation of deviations, AMY: average daily milk yield.

Indicator	Number of cows	Mean	SD	Min	Max	CV (%)
RawVar	2,644	1.4004	0.7999	−1.2193	3.7983	57
LnVar1	2,645	0.4599	0.6857	−1.8190	2.4765	149
lnVar2	2,649	−3.9081	0.7961	−6.5300	−1.2156	20
Skew1	2,660	−0.57	0.9026	−4.3147	2.9202	158
Skew2	2,649	−0.3024	1.053	−5.3467	5.2933	348
r_auto_1	2,666	0.3154	0.2493	−0.4178	0.9157	79
r_auto_2	2,666	0.3100	0.2494	−0.4189	0.9179	80
AMY	2,670	9.1916	3.7006	0.5591	21.2462	40

1 and 2 denote that the resilience indicator was calculated from actual and standardized deviations, respectively.

### 3.2 Factors affecting resilience indicators

Summary statistics from the least squares analyses of variance for resilience data are presented in Table 3. The effect of breed group on general resilience of animals was different depending on the resilience indicators used. Three indicators based on variance (RawVar, LnVar1, and LnVar2) showed that animals with ≤50% dairy genes in their genetic makeup (GC1) had the lowest indicator values followed by those with >50 to ≤87.5% (GC2) and more than 87.5% (GC3) *B. taurus* genes in that order. This indicates that the degree of resilience tended to increase with a decrease in the percentage of taurine genes in the genetic makeup of the animal when these three are utilized as resilience indicators. GC1 animals had a significantly better degree of general resilience than GC2 animals and GC2 animals had a better degree of resilience than GC3 animals. Similarly GC1 cows had lower r_auto_1 signifying a better degree of resilience than GC3 cows (*p* < 0.05). Skew1, Skew2, and r_auto_2 did not detect marked differences in the degree of resilience among animals in different genotypic classes.

**TABLE 3:** Some factors influencing resilience indicators, number of animals, and least square mean of resilience indicator (LSM, SE in parentheses) at each factor level.

Variable, and level	RawVar		LnVar1		LnVar2		Skew1		Skew2		Auto1		Auto2	
	N	LSM (SE)	N	LSM (SE)	N	LSM (SE)	N	LSM (SE)	N	LSM (SE)	N	LSM (SE)	N	LSM (SE)
Breed group														
GC1	902	0.25 (0.09)^a^	904	−0.46 (0.08)^a^	914	−3.99 (0.07)^a^	921	−0.72 (0.12)^a^	915	−0.39 (0.14)^a^	926	0.06 (0.03)^a^	927	0.07 (0.03)^a^
GC2	598	0.36 (0.1)^a^	598	−0.36 (0.09)^a^	596	−4.09 (0.08)^b^	598	−0.6 (0.14)^a^	597	−0.47 (0.16)^a^	598	0.08 (0.03)^ab^	598	0.08 (0.03)^a^
GC3	1,144	0.69 (0.1)^b^	1,143	−0.08 (0.08)^b^	1,139	−3.89 (0.07)^c^	1,141	−0.73 (0.14)^a^	1,137	−0.55 (0.16)^a^	1,142	0.1 (0.03)^b^	1,141	0.09 (0.03)^a^
Herd environment level														
SAA	670	0.38 (0.09)^a^	672	−0.4 (0.08)^a^	678	−3.34 (0.08)^a^	690	−0.4 (0.13)^a^	678	−0.13 (0.15)^a^	695	0.16 (0.03)^a^	696	0.15 (0.03)^a^
SAP	393	0.68 (0.09)^b^	393	−0.19 (0.08)^b^	391	−4.43 (0.08)^b^	391	−1.23 (0.13)^b^	391	−1.11 (0.15)^b^	392	0.16 (0.03)^a^	392	0.17 (0.03)^a^
SH	1,581	0.26 (0.13)^a^	1,580	−0.34 (0.11)^a^	1,580	−4.24 (0.11)^b^	1,577	−0.42 (0.17)^a^	1,580	−0.16 (0.2)^a^	1,579	−0.07 (0.03)^b^	1,578	−0.08 (0.04)^b^

Significant differences at *p* < 0.05 have been shown using different letters. Similar letters indicate differences are not significant.

The environment where the animals were reared influenced all resilience indicators, hence the level of general resilience. For RawVar and LnVar1, animals performing in semi-arid arable (SAA) had the lowest variance, thus were the most resilient and those in a semi-humid environment had the highest variance, hence were the least resilient to the disturbances within their respective environments (*p* < 0.05). According to Skew1 and Skew2, animals in the two semi-arid environments had higher skewness of deviations than those performing in semi-humid environment (*p* < 0.001). However, no significant difference was observed in both skewness of deviations indicators between animals in the two semi-arid environments. This implies that animals reared in the two semi-arid environments had at the population level the same degree of resilience but were significantly more resilient to the disturbances within their respective environments than those reared in a semi-humid environment when Skew1 and Skew2 are used as resilience indicators. Animals in semi-arid arable agroecological zone had the lowest r_auto_1 and r_auto_2 and thus were the most adapted to their environment (*p* < 0.001) according to these indicators. The two indicators did not reveal a significant difference between the degree of resilience of herds in semi-arid pasture-based and semi-humid environments. Contrary to all other indicators, LnVar2 found that animals in the semi-humid agroecological zone had the lowest variance, hence the highest level of adaptability to the disturbances therein (*p* < 0.001). In addition, it indicated that animals in the semi-arid arable agroecological zone had significantly lower variance and were consequently more adapted to their environment than those in the semi-arid pasture-based agroecological zone (*p* < 0.001).

### 3.3 Genetic parameters of resilience indicators

Variance components, heritability estimates, and genetic coefficient of variation of the resilience indicators are reported in Table 4. The heritability estimates of resilience indicators were generally low. Of all indicators, those based on variance had the highest heritabilities that were all significant from zero (*p* < 0.05). Raw variance, which is the variance of lactation milk yield, had the highest heritability estimate of 0.27. Log-transformed variance based on actual deviations (LnVar1) and standardized deviations (LnVar2) had heritabilities of 0.19 and 0.17, respectively. The heritabilities of r_auto_1 and r_auto_2 were 0.05 and 0.07, respectively, and were significantly different from zero (*p* < 0.05). The heritability estimate of Skew1 was the lowest and was not significantly different from zero, whereas that of Skew2 was 0.05 and tended to be significant (*p* = 0.08). The genetic coefficients of variation of all resilience indicators were greater than 0.1. Skew2 had the highest GCV of 0.7 which might have been inflated by a close-to-zero mean of this indicator. Therefore, most resilience indicators based on the fluctuations in milk yield are heritable and show genetic variability.

**TABLE 4:** Variance components (additive genetic variance $\left(\sigma_{a}^{2}\right.$ ), error variance ( $\sigma_{e}^{2}$ ), phenotypic variance ( $\sigma_{p}^{2}$ ), heritabilities ( $h^{2}$ ) and genetic coefficients of variation (GCV)) of the resilience indicators with standard error in brackets from the univariate analyses.

Trait	$\sigma_{a}^{2}$	$\sigma_{e}^{2}$	$\sigma_{p}^{2}$	GCV	$h^{2}$	*p*-Value of $h^{2}$
LnVar1	0.057 (0.013)	0.242 (0.012)	0.299 (0.009)	0.24	0.19 (0.04)	<0.0001
r_auto_1	0.001 (0.001)	0.029 (0.001)	0.031 (0.001)	0.12	0.05 (0.03)	0.0487
Skew1	0.014 (0.018)	0.711 (0.027)	0.725 (0.021)	0.2	0.02 (0.03)	0.3833
LnVar2	0.047 (0.011)	0.225 (0.011)	0.272 (0.008)	0.22	0.17 (0.04)	<0.0001
r_auto_2	0.002 (0.001)	0.029 (0.001)	0.031 (0.001)	0.15	0.07 (0.03)	0.0052
Skew2	0.045 (0.03)	0.904 (0.037)	0.949 (0.028)	0.7	0.05 (0.03)	0.0822
RawVar	0.102 (0.019)	0.279 (0.016)	0.381 (0.012)	0.32	0.27 (0.05)	<0.0001

1 Indicates that the resilience indicator was calculated from actual deviations of milk yield from expected yield, and 2 indicates that the indicator was defined from standardized deviations.

Phenotypic and genetic correlations between different resilience indicators are presented in Table 5. RawVar had a strong positive genetic correlation with LnVar1 (r = 0.85) signifying that they might be genetically more similar traits. Besides, the use of actual and standardized deviations to define both autocorrelation and skewness of deviations indicators yielded highly similar resilience traits as evidenced by strong positive genetic correlations between them (0.94 and 0.81 for autocorrelation and skewness of deviations, respectively). Similar observations were made for phenotypic correlations between r_auto_1 and r_auto_2 (0.91) as well as Skew1 and Skew2 (0.76). There seemed to be a moderate negative correlation between autocorrelation and skewness of deviations. This means that a resilient animal declared so by using autocorrelation of deviations is likely to be defined as resilient using skewness of deviations. LnVar1 and LnVar2 had significant negative genetic correlation, signifying that they are genetically different traits (0.29). However, there was a significant moderate positive phenotypic correlation between them (0.47). Phenotypic and genetic correlations among all other resilience indicators were weak to moderate.

**TABLE 5:** Phenotypic (below diagonal) and genetic correlations (above diagonal) with standard errors (in parentheses) of the resilience indicators.

	RawVar	LnVar1	LnVar2	r_auto_1	r_auto_2	Skew1	skew2
**RawVar**		0.85 (0.07)*	−0.19 (0.14)	0.42 (0.26)	0.21 (0.22)	−0.66 (0.6)	−0.19 (0.27)
**LnVar1**	0.57 (0.01)*		−0.29 (0.14)*	0.08 (0.26)	−0.06 (0.22)	−0.81 (0.65)	−0.51 (0.27)
**LnVar2**	0.19 (0.02)*	0.47 (0.02)*		0.13 (0.27)	0.08 (0.24)	0.24 (0.4)	0.33 (0.26)
**r**_**auto**_ **1**	0.09 (0.02)	0.24 (0.02)*	0.24 (0.02)*		0.94 (0.07)*	−0.92 (0.5)*	−0.99 (0.58)
**r**_**auto**_ **2**	0.07 (0.02)	0.2 (0.02)*	0.22 (0.02)*	0.91 (0)*		−0.96 (0.47)*	−0.89 (0.25)*
**Skew1**	−0.11 (0.02)	−0.24 (0.02)*	0.05 (0.02)	0.00 (0.02)	−0.02 (0.02)		0.81 (0.27)*
**skew2**	−0.04 (0.02)	−0.11 (0.02)	0.22 (0.02)*	0.02 (0.03)	−0.02 (0.02)	0.76 (0.01)*	

1 Signifies that the resilience indicator was calculated from actual deviations of milk yield from expected yield and 2 shows that the indicator was based on standardized deviations. Asterisk signifies significance at *p* < 0.05.

### 3.4 Genetic parameters of longevity and average milk yield

Two longevity traits, productive lifespan (PLS) and herd life (HL), had heritability estimates of 0.1 and 0.08, respectively, which were both significantly different from zero. They had low GCV of 0.008 and 0.004, respectively, indicating a low genetic variability (Table 6). As expected, the two longevity traits were strongly positively correlated, showing that they contain a common genetic variation (r_p_ = 0.99 and r_g_ = 0.95). Average test-day milk yield, on the other hand, had an estimated heritability of 0.43 and a genetic coefficient of variation of 0.15, implying that it is heritable with high genetic variability.

**TABLE 6:** Variance components (additive genetic variance $\left(\sigma_{a}^{2}\right.$ ), error variance ( $\sigma_{e}^{2}$ ), phenotypic variance ( $\sigma_{p}^{2}$ ), heritabilities ( $h^{2}$ ) and genetic coefficients of variation (GCV)) of average milk yield (AMY), productive lifespan (PLS), and herd life (HL) from the univariate analyses.

Trait	$\sigma_{a}^{2}$	$\sigma_{e}^{2}$	$\sigma_{p}^{2}$	GCV	$h^{2}$	*p*-Value of $h^{2}$
LPS	0.457 (0.224)	2.904 (0.232)	3.36 (0.158)	0.01	0.14 (0.07)	0.0154
HL	0.336 (0.203)	2.907 (0.22)	3.243 (0.151)	0.005	0.1 (0.06)	0.0482
AMY	1.919 (0.242)	2.536 (0.181)	4.455 (0.146)	0.15	0.43 (0.04)	<0.0001

The phenotypic and genetic correlations of resilience indicators and productive lifespan, herd life, and average test-day milk yield are presented in Table 7. Phenotypic correlations between all resilience indicators and two longevity traits were low and non-significant. Similarly, genetic correlations between longevity traits and resilience indicators were negligible, except for LnVar1 and LnVar2. A negative genetic correlation was observed between both LnVar1 and LnVar2 and longevity traits. This shows that more resilient animals have better longevity. RawVar and LnVar1 had positive significant genetic and phenotypic correlations with AMY. This implies that for LnVar1 and RawVar indicators, more resilient animals produce less lactation milk yield and *vice versa*. LnVar2 on the other hand, had significant negative genetic and phenotypic correlations with average test-day milk yield, which means that a high average test-day milk yield is expected of a resilient animal. All other resilience indicators had low nonsignificant phenotypic and genetic correlations with average milk yield.

**TABLE 7:** Phenotypic (r_p_) and genetic (r_g_)correlations with standard errors (in parentheses) of the resilience indicators with average milk yield, productive lifespan, and herd life.

Resilience indicator	Productive lifespan		Herd life		Average milk yield	
	r_g_	r_p_	r_g_	r_p_	r_g_	r_p_
RawVar	−0.19 (0.26)	0.04 (0.03)	−0.29 (0.28)	0.02 (0.04)	0.71 (0.07)*	0.4 (0.02)*
LnVar1	−0.48 (0.25)*	−0.04 (0.04)	−0.52 (0.27)*	−0.04 (0.04)	0.72 (0.08)*	0.41 (0.02)*
LnVar2	−0.42 (0.26)*	−0.05 (0.04)	−0.42 (0.29)*	−0.05 (0.04)	−0.66 (0.08)*	−0.41 (0.02)*
r_auto_1	−0.29 (0.48)	0.01 (0.03)	0.18 (0.51)	0.01 (0.03)	−0.14 (0.21)	−0.01 (0.02)
r_auto_2	−0.37 (0.42)	0.01 (0.03)	−0.31 (0.46)	0.01 (0.03)	−0.2 (0.17)	−0.02 (0.02)
Skew1	0.08 (0.68)	−0.03 (0.04)	0.06 (0.72)	−0.03 (0.04)	−0.61 (0.54)	−0.1 (0.02)
skew2	0.44 (0.47)	−0.01 (0.03)	0.63 (0.52)	−0.02 (0.03)	−0.59 (0.23)*	−0.17 (0.02)*

Asterisk signifies significance at *p* < 0.05.

## 4 Discussion

This study tested the potential for using variance, autocorrelation, and skewness of deviations in milk yield to measure resilience of the dairy cows performing in a tropical environment of sub-Saharan Africa. This is the first study aimed at estimating the genetic parameters of these resilience indicators for dairy animals in SSA. First parity milk records were used in calculating the resilience indicators for several reasons: i) first parity lactation milk yield is good at predicting lifetime resilience of dairy cows (Adriaens et al., 2020); ii) Early determination of general resilience of animals allows farmers to make quicker and better informed breeding and management decisions much earlier and thereby reduce resources wastages associated with the continued keeping of non-resilient animals iii) Fitness related traits such as general resilience are normally under stronger directional selection than non-fitness traits (Mousseau and Roff, 1987; Falconer and Mackay, 1996; Sheldon and Merilä, 1999). Thus, use of milk records from higher parities might deflate heritability estimates because as non-resilient animals that fail to survive up to the subsequent calving are culled (naturally selected against), genetic variation within the population under study is eroded.

A fourth-order polynomial regression was used to predict lactation curves since it has a smaller risk of being too flexible and has additional parameters as compared to other trend estimation methods such as Wilmink lactation curve (Macciotta et al., 2005; Poppe et al., 2020). The use of quantile regression instead of classical linear regression to estimate the lactation curves made the curves less sensitive to drops in milk yield and thus closer to the potential curves in the absence of disturbances. This is because quantile regression models estimate the conditional median or other quantiles of milk yield (and not the conditional mean) given certain values of days in milk (Koenker et al., 2005). Besides, it was found to be the best method to fit the expected lactation curves for defining resilience indicators (Poppe et al., 2020). The choice of quantile is critical for a good prediction of lactation curve considering that disturbances in the environment lower milk yield produced. A quantile higher than 0.5 is recommended because it makes drops in milk yield values have less influence on the predicted milk yield curve than do high milk yield values (Koenker et al., 2005; Poppe et al., 2020).

Generally, dairy cattle with a higher percentage of taurine genes were less resilient. Cows with less than 87.5% were more resilient than those with over 87.5% *Bos taurus genes*. Zebu cattle are reportedly adapted to the SSA production environment and its disturbances (Renaudeau et al., 2012; Mwai et al., 2015; Wilson, 2018). Our study has therefore confirmed previous reports that presence of Zebu genes in the genetic composition of a crossbred cow bestows a resilience advantage in coping with local production conditions and the associated disturbances within these environments (Oloo et al., 2022b). There was no significant (*p* < 0.05) difference in general resilience between cows that had 0%–50% and those with >50–87.5% *B. taurus* genes. It was even surprising that for log-transformed variance of standardized deviations, cows with >50–87.5% *B. taurus* genes were more resilient. This suggests that crossbred cattle with dairyness between 50% and 87.5% might be the ideal genotypes for the sub-Saharan African environment since they are capable of producing more milk than pure Zebu cattle (Mujibi et al., 2019; Ojango et al., 2019) and at the same time able to withstand more stressful environmental disturbances.

Dairy cows performing in semi-arid zones had a higher degree of general resilience in this study. All indicators showed that animals in the two semi-arid arable agroecological zones were more resilient than those performing in a semi-humid environment. Semi-arid regions have many disturbances, such as low rainfall, prolonged periods of drought, high temperatures, and shortages of feed and pastures. Continuous exposure of animals performing in semi-arid environments to such disturbances could have possibly activated their inherent regulatory pathways and enhanced their ability to withstand environmental stressors in the long run (Colditz and Brad, 2016). The environmental stimulation and experiences in semi-arid regions helped the animals to acquire genetic/biological adaptation and evolved to survive in adverse conditions (Parsons, 1994; Hansen, 2004; Gaughan et al., 2009). It is also possible that both artificial (earlier culling of less adaptable animals) and natural (i.e., loss through death, of cows in early life, are not able to cope with local stressful environments) selection were at work in semi-arid zones hence, only more resilient animals lived long enough to calve and produce milk in these conditions.

Resilience indicators had low to moderate heritabilities that were, in most cases, significantly different from zero. The log-transformed variance of lactation milk yield had the highest heritability estimate, which was comparable to those previously reported in other studies (Elgersma et al., 2018; Poppe et al., 2020). Of indicators derived from deviation of milk yield, log-transformed variance (LnVar) of deviation had the highest heritabilities that were comparable to those initially reported (Poppe et al., 2021a; Poppe et al.2021b; Poppe et al. 2020). The genetic coefficient of variation of all indicators based on variance (log-transformed) ranged from 0.22 to 0.32 which was within the range of previously reported estimates based on variation of different traits in different livestock species (Mulder et al., 2013; Rönnegard et al., 2013; Vandenplas et al., 2013; Sell-Kubiak et al., 2015; Elgersma et al., 2018; Berghof et al., 2019a). This means that genetic improvement based on selection of log-transformed variance as a general resilience indicator is possible under SSA conditions. Autocorrelation of deviation based on two methods had significant but low heritabilities that were also comparable to those reported by Poppe et al. (2020, 2021a). Similar to other studies (Berghof et al., 2019a; Poppe et al., 2020; Poppe et al., 2021b), skewness of deviation had low and nonsignificant heritabilities.

The use of actual deviation and standardized deviation to define indicators yielded identical traits except in LnVar. A strong positive genetic correlation between skew of deviation and skew of standardized deviation suggests a similarity between these two traits. This similarity could be attributed to the fact that the sign or direction of deviation does not change whether actual or standardized deviation is used. Therefore, the final value an animal gets for skewness will vary depending on how deviation is defined, but the direction will not change. Similarly, indicators for autocorrelation of deviation were highly correlated, indicating that they are similar. This might be related to the generally low to average milk production and more flat lactation curves observed for animals in this study. Indeed, it has been reported that the milk yield of dairy animals performing in SSA is considerably low in comparison to those performing in the temperate world (Galukande et al., 2013; Ojango et al., 2017). Due to their low production profile, expected milk yield values for two consecutive test days, which is used as a base when calculating standardized deviation, were almost equal. As a result, a high correlation was expected between the values of actual and standardized deviations, which translated to their genetic correlation. The genetic correlation between r_auto_1 and r_auto_2 is likely to get lower as the differences in expected milk yield between two consecutive test days increase, as in the case of high-producing taurine animals.

However, for LnVar indicators, there was a negative correlation between the two traits, indicating that they are dissimilar. This is because variance considers how different the values are from the mean. Being ratios, standardized deviations are smaller with narrower distribution and hence have lower variance as compared to actual deviations. Besides, animals with equal deviations have different standardized deviations unless their expected milk yield is also equal. We found a strong positive correlation between the variance of actual deviation and the variance of lactation milk yield, which is contrary to previous findings (Poppe et al., 2020; Poppe et al., 2021a; Poppe et al. 2021b). This could probably be due to the low production profiles of the cows under study, which resulted in flat modeled lactation curves. Besides, proportionately high, and negative permanent environmental variances associated with these cows, especially given the usual below-average management levels to which such animals are exposed during an earlier stage in life, could have contributed to flattened lactation curves. Thus, most of the expected milk yield values across the lactation were closer to the average test-day milk yield, which made the variance of deviations and that of lactation milk yield more alike hence the higher correlation.

Genetic correlations between different indicators were negligibly low, except between autocorrelation and skewness of deviations. This perhaps implies that either some of the indicators are not good predictors of general resilience or different resilience indicators capture different aspects of resilience (Berghof et al., 2019a; Berghof et al., 2019b). A negative moderate correlation observed between autocorrelation and skewness of deviations indicates that a resilient animal based on autocorrelation is likely to be resilient based on skewness of deviation. Biologically, an animal with a fast recovery rate from disturbance (low autocorrelation) is likely to have a symmetric distribution of milk yield deviations from its lactation curve. A study by Poppe et al. (2020) reported almost similar results of correlation between the two indicators as those reported here. To holistically improve general resilience, all known aspects of resilience need to be captured. Therefore, different resilience indicators should be combined in a multi-trait index, and improvements in the predictive ability of this index to measure resilience tested against individual indicators.

Both herd life and longevity had heritability estimates that were comparable to those reported in the literature (Ghaderi-Zefrehei et al., 2017; Imbayarwo-Chikosi et al., 2018; Oliveira et al., 2020). Dairy cows with a long productive life span generally have few health problems, good reproductive performance, and efficient and consistent milk production (Adriaens et al., 2020), therefore, resilient animals are expected to have greater longevity. Analysis of the relationship between resilience indicators and longevity traits showed that only LnVar1 and LnVar2 had a significant negative correlation with longevity. This means that resilient animals had a long productive life span and herd life. Therefore, variance of actual deviations and variance of standardized deviations might indicate general resilience in this sense. These findings agree well with earlier reports (Elgersma et al., 2018; Poppe et al., 2020; Poppe et al., 2021a). Phenotypic correlations between all indicators and longevity traits were very low and non-significant but in the same direction as genetic correlations except for autocorrelation and skewness of deviations indicators. This shows that general resilience, like other fitness-related traits, is highly influenced by the environment and/or has a high non-additive variation (Sheldon and Merilä, 1999).

Although not significant, correlations of indicators based on autocorrelation and skewness of deviations indicators with longevity traits were in the expected direction. The lack of significant correlations between autocorrelation and skewness of deviations with longevity could be attributed to the dataset and/or the properties of these indicators. In SSA dairy systems, data recording is normally manual hence, daily milk yield records are usually not available in most cases. Autocorrelation of deviation is expected to indicate the duration of (rate of recovery from) a disturbance (Berghof et al., 2019b). This study used a minimum of ten observations per individual. Sparsely distributed observations in our dataset might have failed to capture information about the recovery rate from disturbance adequately. Skewness of deviations is more sensitive to erroneous milk yield values and a single outlier could greatly affect it (Berghof et al., 2019a; Berghof et al., 2019b; Poppe et al., 2020). However, it was impossible to remove all outliers from the data because doing so would be too stringent and would also remove records that were informative about resilience. Besides, productive longevity is more of a robustness than a resilience trait, as it is an integration over time of an animal’s cumulative ability to overcome the environmental challenges it has faced throughout life (Friggens et al., 2017). Therefore, these two indicators may not be good measures of herd life and productive longevity.

Three resilience indicators, variance of milk yield, variance of actual deviations, and variance of standardized deviations had significant correlations with milk yield. The former two showed a negative correlation suggesting that more resilient animals have low average test-day milk yield as previously reported by Elgersma et al. (2018) and (Poppe et al., 2020; Poppe et al., 2021a). This was expected because the range in daily milk yield of low-producing animals is normally low hence they have a low variance of lactation milk yield and actual deviations as compared to high-producing animals. However, it is not always the case that low-producing animals are more resilient. A lack of resilience itself can make an animal produce well below its potential, especially when performing in a more challenging environment (Berghof et al., 2019b). On the other hand, the variance of standardized deviation had a positive correlation with average milk yield, denoting that more resilient animals in these environments had higher average milk yield and *vice versa*. Indeed, least squares analyses of variance for average test-day milk yield in this study found no significant difference between 50% and 87.5% (more resilient) and >87.5% (less resilient) *B. taurus* animals. This is possible biologically because resilient animals have increased adaptability potential and their performance is either unaffected or less affected by the disturbances in the environment (Mulder et al., 2013; Colditz and Brad, 2016). As such, resilient animals tend to have a production profile that is closer to their optimal performance levels as compared to non-resilient animals. However, this observation is environment-specific and it does not necessarily mean that resilient animals are high milk producers. These findings suggest that the variance of standardized deviations does not erroneously categorize low-producing animals as resilient. Consequently, it may serve as a more reliable measure for assessing general resilience of dairy animals in SSA.

## 5 Conclusion

This study assessed the potential of using log-transformed variance, skewness, and autocorrelation of actual and standardized deviations in milk yield to quantify general resilience of dairy animals in sub-Saharan Africa. Generally, these indicators showed that the presence of *Bos indicus* genes in the genetic makeup of the animals improved their resilience capabilities to environmental disturbances. Exposure of animals to a wide range of disturbances in semi-arid regions improved their resilience capacity. The study further demonstrated that log-transformed variance and autocorrelation of actual and standardized deviations were heritable, although their heritabilities ranged from low to moderate. These resilience indicators captured different aspects of general resilience as shown by a lack of significant correlation between them. The use of actual and standardized deviations resulted in two genetically different variance of deviations traits. Both indicators based on variance of deviations had negative correlations with longevity traits. Variance of actual deviation showed that resilient animals produce low average milk yield and *vice versa*. Nonetheless, variance of standardized deviation displayed a positive genetic correlation with average milk yield implying that resilient animals produce higher average milk yield. Of these indicators, variance of standardized deviations seems a better indicator for assessing resilience as it is not biased towards low-producing animals and is genetically associated with better longevity as well as higher average test-day milk yield. This research is vital for the improvement of dairy productivity through breeding for resilience especially in SSA. Other researched methods of quantifying resilience of animals need to be tested in SSA and a selection index for general resilience developed from promising indicators.

## Data Availability

The raw data supporting the conclusion of this article will be made available by the authors, without undue reservation.
